# Design Features Associated With Engagement in Mobile Health Physical Activity Interventions Among Youth: Systematic Review of Qualitative and Quantitative Studies

**DOI:** 10.2196/40898

**Published:** 2023-03-06

**Authors:** Ayla Schwarz, Laura H H Winkens, Emely de Vet, Dian Ossendrijver, Kirsten Bouwsema, Monique Simons

**Affiliations:** 1 Department of Social Sciences, Chair Group Consumption & Healthy Lifestyles Wageningen University & Research Wageningen Netherlands

**Keywords:** systematic review, youth, physical activity, design features, engagement, mHealth, mobile health, mobile phone

## Abstract

**Background:**

Globally, 81% of youth do not meet the physical activity (PA) guidelines. Youth of families with a low socioeconomic position are less likely to meet the recommended PA guidelines. Mobile health (mHealth) interventions are preferred by youth over traditional in-person approaches and are in line with their media preferences. Despite the promise of mHealth interventions in promoting PA, a common challenge is to engage users in the long term or effectively. Earlier reviews highlighted the association of different design features (eg, notifications and rewards) with engagement among adults. However, little is known about which design features are important for increasing engagement among youth.

**Objective:**

To inform the design process of future mHealth tools, it is important to investigate the design features that can yield effective user engagement. This systematic review aimed to identify which design features are associated with engagement in mHealth PA interventions among youth who were aged between 4 and 18 years.

**Methods:**

A systematic search was conducted in EBSCOhost (MEDLINE, APA PsycINFO, and Psychology & Behavioral Sciences Collection) and Scopus. Qualitative and quantitative studies were included if they documented design features associated with engagement. Design features and related behavior change techniques and engagement measures were extracted. Study quality was assessed according to the Mixed Method Assessment Tool, and one-third of all screening and data extraction were double coded by a second reviewer.

**Results:**

Studies (n=21) showed that various features were associated with engagement, such as a clear interface, rewards, multiplayer game mode, social interaction, variety of challenges with personalized difficulty level, self-monitoring, and variety of customization options among others, including self-set goals, personalized feedback, progress, and a narrative. In contrast, various features need to be carefully considered while designing mHealth PA interventions, such as sounds, competition, instructions, notifications, virtual maps, or self-monitoring, facilitated by manual input. In addition, technical functionality can be considered as a prerequisite for engagement. Research addressing youth from low socioeconomic position families is very limited with regard to engagement in mHealth apps.

**Conclusions:**

Mismatches between different design features in terms of target group, study design, and content translation from behavior change techniques to design features are highlighted and set up in a design guideline and future research agenda.

**Trial Registration:**

PROSPERO CRD42021254989; https://tinyurl.com/5n6ppz24

## Introduction

### Background

Physical activity (PA) in youth is associated with a variety of health benefits [1], including physical [2] and mental health benefits [3]. Despite the health benefits, 81% of youth (ie, those in childhood and adolescence) globally do not meet the PA guidelines [4,5] of daily 60 minutes of moderate- to vigorous-intensity physical activity (MVPA) and vigorous activities 3 days per week [1]. In Europe, only 19% of adolescents comply with the MVPA guidelines, and higher family affluence is associated with higher levels of MVPA [6]. Moreover, PA in youth is reported to track into adulthood, underlying the importance of promoting PA in the youth [7,8]. Also, the youth of families with a low socioeconomic position (SEP) are less likely to meet the recommended PA guidelines [9,10]. Therefore, effective and socially acceptable PA interventions are needed [11], especially among youth of families with a low SEP. SEP is often measured in terms of education (attainment), income, and occupation status, which are often interrelated and related to the social and economic resources available [12].

Mobile health (mHealth) tools such as smartphone apps can be cost-effective [13] in changing total PA [14,15] and daily steps [14]. mHealth interventions are preferred by youth over traditional in-person approaches [16-18] and are in line with preferences of youth, with regard to multimedia formats (ie, text, sound, and video) [19]. Mobile devices and apps have gained popularity in the daily life of increasingly younger age groups of youth, starting at the age of 3 or 4 years already [20-24]. Families of young children, especially those with a low-SEP background, indicate concern about inappropriate content [20], which might suggest that appropriate content might rather be supported by guardians. Appropriate content may refer to different forms of app use that might not necessarily increase current screen time use, as the design of the mHealth intervention may have considered a limited use time frame, only stimulating sporadic screen time (eg, integration to the direct physical environment to stimulate children to go outside and be physically active instead of the requirement of using the digital tool when being physically active) or stimulating the co-use of young children and guardians [25]. Earlier systematic reviews indicated that currently, foremost, adults are included in mHealth effectiveness studies, and limited research has been conducted among youth [26]. People with high SEP compared with people with low SEP report foremost positive health effects of the same digital intervention [27]. Apps are primarily designed for people with high SEP, and people with low SEP are often reported as being difficult to reach [28]. In contrast to mHealth apps, youth from low-SEP families engage in more screen time [29] and are more involved on the web [30] and active [31] gaming. This suggests that approaching youth with low SEP via apps (eg, screen and games) can be useful and beneficial, yet other ingredients (eg, tailoring) are needed to reach them effectively in mHealth apps [32].

Despite the promise of digital or mHealth interventions in promoting PA [26], a common challenge is to engage users in the long term [14] or effectively [26]. With regard to including people with a low-SEP background in research, studies identified challenges in reaching the target group [33] and engaging them in research [28] and in the digital health intervention [34]. In addition, eHealth and mHealth studies focus on a general target group and often do not research subgroups (ie, low-SEP groups) [35].

Engagement refers to the involvement and motivation of the user in the intervention [36]. It refers to “(1) the extent (e.g., amount, frequency, duration, depth) of usage and (2) the subjective experience characterized by attention, interest and affect” (ie, enjoyment) [37]. Attention refers to the degree of focus or absorption versus distraction in the intervention. Interest refers to feelings of interest or fascination versus boredom with the intervention. Enjoyment reflects the enjoyable experience of fun or pleasure versus frustration and annoyance while using the intervention [37,38]. Engagement can contribute to the overall effectiveness of digital interventions [26,39], meaning that to be effective, it is important that the user experiences a sufficient degree of engagement. The extent of use or dose is less predictive of engagement than the subjective experience to achieve the intended outcomes of the intervention (ie, effective engagement) [40]. This means that frequently engaging with the intervention is not always required to be effectively engaged with the intervention or the behavior eventually.

Currently, engagement is still not commonly reported in terms of use data (ie, extent) [26] and experience data (ie, subjective engagement) [41]. To improve intervention exposure and related intervention efficacy, it is important to better understand the design features (ie, active ingredients of an app that are in best cases informed by theory and translated into design, often referred to as “persuasive features,” features, and elements) contributing to engagement [26]. A review of commercial apps indicated low engagement scores and suggested investigating features that improve engagement with an app among youth [42], as the features may differ with those studied in adults [37]. Earlier studies reported a common decline in app use over time and called for investigating features that contribute to intervention uptake and engagement [14,26] also among particular subgroups, such as people with a low-SEP background [27,32,43,44]. For example, research among low-SEP groups indicated that frustration with particular design features and navigations (eg, data log) hampered app use [45].

When designing mHealth interventions, several active ingredients, otherwise indicated as behavior change techniques (BCTs), are applied and translated to app features to foster behavior change. In addition to low engagement scores, existing reviews reveal that a limited number of BCTs, such as instructions, encouragement, rewards, and feedback on performance, which are essential components of interventions to promote behavior change [46], are currently applied in mHealth apps [42]. BCTs can be directly translated into gamification or app features (eg, goal-setting translated into challenges), here referred to as design features [47]. Studies suggest that applying either a larger number of BCTs [42]—particular BCTs (eg, self-monitoring, feedback, goal-setting, rewards, reminders, and social support under certain circumstances) or a particular combination of BCTs (eg, problem-solving and rewards) [48]—may contribute to engagement in digital interventions [44,47,48].

### Objective

To inform the design process of future mHealth tools, it is important to investigate the design features that can yield effective user engagement. Research has demonstrated that for adults, user guidance, well-designed reminders, self-monitoring, positive feedback, rewards (eg, lottery), goal-setting, personalization, social networking, and health message framing [43,44,49] were associated with higher user engagement. However, most studies included adult samples, targeted mental health instead of PA among adolescents, or did not focus on low-SEP groups [50-52]. To our knowledge, no systematic review that primarily seeks to identify design features in mHealth PA intervention among youth, especially youth from low-SEP families that have been associated with engagement, has yet been conducted. Therefore, this systematic review aimed to identify which features are associated with engagement in mHealth PA interventions among youth who were aged between 4 and 18 years. This could help inform app developers and (digital) behavior change intervention researchers to better integrate engagement during the design process of mobile behavioral change interventions.

## Methods

### Systematic Review

The research protocol of the review was registered with the International Prospective Register of Systematic Reviews (PROSPERO; #CRD42021254989). Reporting complied with the PRISMA (Preferred Reporting Items for Systematic Reviews and Meta-Analyses; Multimedia Appendix 1 [53]) statement [54].

### Search Strategy

A systematic search was conducted on June 2, 2021, updated on June 24, 2022, and included the following databases: EBSCOhost (MEDLINE, APA PsycINFO, and Psychology & Behavioral Sciences Collection) and Scopus. The search terms that included synonyms relating to engagement and the population (youth), the intervention (mHealth), and outcome (PA) were informed by earlier reviews [37,55,56] and were eventually reviewed by an academic librarian. Multimedia Appendix 2 provides the complete search terms and synonyms used.

### Eligibility Criteria

Quantitative, qualitative, and mixed methods studies were included in the review, as engagement is an emerging field of research, and the aim of this review was to gain a broad overview of features that are considered engaging. Available full-text research articles or conference papers were eligible in case they (1) described the development (ie, user-testing) or evaluation of an mHealth intervention (or more broadly the use of healthy lifestyles as long as specific information about PA could be retrieved), designed for PA promotion; (2) reported on the extent of use or subjective experience; (3) reported an association (ie, relation of design features with engagement, either assessed qualitatively and referred to “self-perceived association” or quantitatively referred to “association”); (4) included an intervention that was designed for healthy youth who were aged between 4 and 18 years (in line with Dutch PA guidelines [57] and in line with the increasing popularity of mobile devices in the daily life of [increasingly younger] youth) [20-24] or if the mean sample age was within this range; (5) were written in English; and (6) were published between 2010 and 2021 (owing to the smartphone use increase among youth, the use of mHealth interventions has been increasing since 2010).

Papers were excluded in case they (1) included a PA intervention that was not mobile based; (2) did not report on the extent of use or subjective experience; (3) did not report an association between a particular intervention feature and engagement; and (4) included either a study population of children who were aged <4 years, adults, or older adults or a population whose mean sample age was not within the range of 4 to 18 years (in line with Dutch national PA recommendation age range). Moreover, editorials, opinion papers, case studies, research protocols, design papers not including any user-testing, book chapters, systematic reviews, or meta-analyses were excluded (Textbox 1).

**Inclusion and exclusion criteria.**

**Inclusion criteria**

- Population
  Youth was defined as individuals who were:
    healthy
    aged between 4 and 18 years or the mean age of sample in the same age range
- Intervention
  Physical activity mobile health interventions that are:
    developed (ie, offered for user-testing) or evaluated
    mobile
    designed for physical activity promotion (or healthy lifestyle, including physical activity)
    designed for the target group of youth (4-18 years old)
- Comparison
  Not applicable
- Outcome
  Drivers of the extent of use or subjective experience with regard to physical activity
- Study type
  Quantitative, qualitative, and mixed methods studies that:
    report on behavioral or subjective engagement
    report a relation or association between the particular feature and engagement
    are original and available full-text research articles or conference paper

**Exclusion criteria**

- Population
  Youth in disease recovery and rehabilitation, experiencing a chronic disease (ie, overweight, obesity, and diabetes)
  Children aged <4 years or mean age not within the age range of 4 to 18 years and adults or older adults
- Intervention
  Physical activity interventions that:
    were still in the early development phase (ie, not offering an app but, for example, only a general discussion about apps, paper mock-ups)
    used different formats than mobile
    only included wearables (eg, smartwatches) without a mobile health app
- Comparison
  Not applicable
- Outcome
  No drivers of the extent of use or subjective experience with regard to physical activity
- Study type
  Quantitative, qualitative, and mixed methods studies that:
    do not report on behavioral or subjective engagement
    do not report a relation or association between the particular feature and engagement, for example, engagement is only described in general without any relation to the particular feature
    are editorials, opinion papers, case studies, research protocols, or design papers not including any user-testing, book chapters, systematic review, or meta-analysis

### Screening and Data Extraction

All the papers identified through the searches were downloaded into Rayyan software (Rayyan Systems Inc), a systematic review software, and duplicates were removed. A multistep strategy was applied. First, titles and abstracts were screened, followed by full-text reading to determine eligibility (DO). One-third of the titles and abstracts and full texts were reviewed by a second reviewer (KB). Interrater reliability for the titles and abstracts and full texts was substantial and moderate (Cohen κ=0.73 and Cohen κ=0.54), respectively. Disagreements regarding the title and abstract and full texts were resolved through discussion between the reviewers (DO and KB). Any disagreement between the 2 reviewers was discussed, and a third reviewer (AS) was consulted if necessary.

Data extraction was completed by both reviewers (DO and KB). Disagreement regarding data extraction was resolved through discussion between the reviewers (DO and KB). Background information (1-3) was extracted by one reviewer (DO), and the rest of the data (4-7) were extracted by two reviewers (DO and KB): (1) publication information (author and year); (2) study information (country and target group [number, percentage of female, percentage of people of low SEP, mean age, and age range]), and 3) app description (name, device, operating system, aim, and theory used; based on the mHealth taxonomy) [58]; (4) engagement measures (engagement measure or definition); (5) presence of BCT and, if present, behavioral change measures (based on the ABACUS scale) [59]; (6) design features (type of feature and 1 feature vs multiple features); and (7) association with engagement (short summary of association of particular features and engagement). The engagement measure of the original individual study was extracted (ie, usability, motivation, enjoyment, and liking) and then coded according to attention (ie, paying attention to mHealth app), interest (ie, feeling interested in mHealth app), and enjoyment (ie, experiencing enjoyment while using it; Textbox 2) [37].

As the researchers of this study were not aware of one framework of design features, 3 (digital) behavior scientist researchers (AS, LW, and MS) developed a coding frame to code all design features based on earlier studies on PA apps; gamification (ie, application of any features [eg, badges, points, avatars etc] in a nongame setting); and game research (ie, full-fledged and rule-based games) [61]. Free codes were created if they did not suit 1 category. Owing to the design process of translating BCTs into design features, obviously, overlap between the features and BCTs occurs (ie, goals vs goal-setting). Design features were coded as interface esthetics, challenges, narrative, levels, feedback, monitoring, customization or personalization, reinforcement, navigation, goals, social, progress, and credibility (Textbox 3).

**Coding scheme of engagement and definitions.**

**Interest**

- Experiencing interest, boredom, fascination, intrigue, or indifference [37]
- Cognitive state that is occurring spontaneously and relates to liking and willful engagement [60]

**Attention**

- Experiencing focus, inattention, absorption, distraction, or mindfulness [37]
- Cognitive state of focused awareness and relates to focalization and concentration [60]

**Enjoyment**

- Experiencing frustration, annoyance, enjoyment, fun, or pleasure [37]
- Relates to the sensory experience and relates to pleasure and activation [60]

**Coding framework for design features and definitions.**

**Interface esthetics**

- Graphics, color, sound, music, and language [55,56]

**Challenges**

- “Time-limited goals or competitions” [55,62-64]

**Narrative**

- Story [54,61,63]
- Levels [55,62,64]
- Feedback [55,56,62,63]

**Monitoring**

- Self-monitoring, monitoring, and tracking of user’s behavior (eg, smartphone sensor and wearable) [55,56,63,65]

**Customization or personalization**

- Assessment of user’s characteristics and provision of personal information and avatars [55,56,62,63]

**Reinforcement**

- Reminders and rewards (badges, points, unlockable content [“access to enhanced functionality or content for accumulating experience or achieving a specific goal”], leaderboard, certificates, medals, cash or gifts, congratulation messages, and updates) [55,56,62-65]

**Navigation**

- Navigation support (menu bar, search bar, etc), instructions, and guidance [55,56]

**Goals**

- “Performance-based measures and targets,” either set by the app or by the user itself [55,63-65]

**Social**

- Social messages (communicating with others), “performance is publicly displayed,” community, competition (compete with each other), and collaboration (work together) [55,56,62-65]

**Progress**

- Progress, achievement, and levels [55,62-64]

**Credibility**

- Absence of advertisement, credible sources or information, privacy policy, and password protection [56,63]

### Quality Assessment

Quality was assessed according to the Mixed Method Assessment Tool [66], and it was not used as a basis for study exclusion but to support the interpretation of results (Multimedia Appendix 3 [67-87]). All the studies were coded by 1 reviewer (DO), and one-third of the studies were double coded (KB). Interrater reliability was substantial (κ=0.63). Any disagreement between the 2 reviewers was discussed, and a third reviewer (AS) was consulted if necessary.

### Synthesis of Results

A systematic narrative format [88] structured around the design features that were associated with engagement was applied. All the features that have been actually tested (ie, user-testing) were reported. In case the same study also provided suggestions by participants to further develop the app (ie, hypothetical design features not tested but suggested), this was marked explicitly as a suggestion. In addition, the presence or absence of BCTs was outlined, indicating whether theories had been applied in the design phase of design features or not. A subgroup analysis was conducted for youth from low-SEP families. The categorization of SEP was derived from the classification of the original studies. Research results were not grouped by the origin of the data (ie, qualitative, quantitative, or mixed methods), but it was indicated explicitly for each research outcome.

## Results

### Description of Included Studies

**Figure 1:**
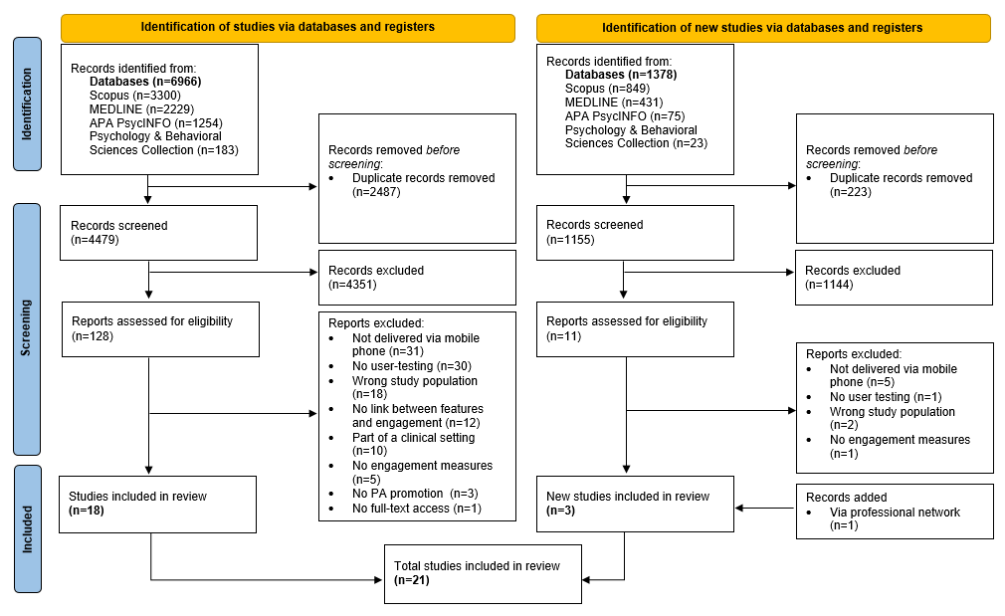
PRISMA (Preferred Reporting Items for Systematic Reviews and Meta-Analyses) flowchart illustrating the inclusion and exclusion of studies. PA: physical activity.

In total, 21 studies were included in this review (Figure 1). The studies originated from different countries (15) and were conducted in Europe (n=10), North America (n=4), Australia (n=3), Asia (n=3), and South America (n=1). These studies were published between 2012 and 2021. Mobile interventions were used and evaluated by youth who were aged between 3 and 18 years. Sample sizes ranged from 5 to 354 youths, with a total sample size of 1443 youths. All the studies retrieved informed consents from parents and youth participants, except for 5 studies that only collected consent from the youth participants alone [67,68] or from the school principal [70], were conducted in parent-child dyads [69], or did not clearly state this [71]. Studies included different engagement measures varying from acceptability, experience, usability, motivation, feasibility, enjoyment, engagement, satisfaction, or interest. When categorizing the measures in terms of interest, attention, or enjoyment, most studies (15/21, 71%) measured solely interest, especially in terms of features that were in general liked by the users. A combination of interest and attention (2/21, 10%), solely attention (1/21, 5%), solely enjoyment (1/21, 5%), or solely engagement (2/21, 10%) was rarely studied. Engagement was mainly measured as the primary outcome in some studies (14/21, 67%). Most studies (12/21, 57%) used a mixed methods design, ranging from posttest prototype studies to quasi-experimental studies to randomized controlled trials, mixed with designs such as qualitative exit interviews, focus groups, or think-aloud studies. In total, 24% (5/21) of studies included a qualitative study design (interview and focus group study), and 19% (4/21) of studies included a quantitative study design (experimental design with quantitative survey). Studies relevant to this review included qualitative data (10/21, 48%), quantitative data (4/21, 19%), or mixed methods data (7/21, 33%) to determine the association between design features and engagement.

On the basis of Mixed Method Assessment Tool [66], the quality of the included studies was mixed. In general, the studies that measured engagement as the primary study outcome had a moderate to high quality, especially scoring high on transparency regarding the data collection methods and analysis. Studies that focused on engagement as a secondary outcome scored rather low on the quality of the study design applicable to measure engagement (eg, including nonvalidated methods such as a number of open exit questions in a larger randomized controlled trial), although the studies scored high on the design (eg, randomized controlled trial) to measure the primary outcome (eg, PA).

### mHealth Interventions

In general, the mobile interventions tested in the included studies (N=21) were either developed for commercial purposes (n=4, 19%) or for study purposes (n=17, 81%). Most of the interventions (14/21, 67%) were designed to improve PA behavior. In addition, 33% (7/21) of studies focused also on additional health behaviors associated with a healthy lifestyle (eg, nutrition or sleep) in addition to PA behavior. The studies included different types of mHealth interventions, ranging from mobile games (10/21, 48%) to mobile apps (8/21, 38%) to mobile text messaging systems (3/21, 14%). Mobile games mainly focused on PA (9/10, 90% studies); included different PA activities (ie, ranging from simple arm movements such as swinging arm to complex full-body movement such as running); and applied different inputs (ie, ranging from manual input to camera data to Bluetooth, GPS, or PA sensor data). Games ranged from short-term activities (eg, push-ups) to long-term gameplay (eg, location-based treasure hunt games). Mobile apps ranged from narrative-driven PA guidance to GPS-tracking information. Mobile text messaging systems especially focused on generating and adapting goal-setting, facilitating self-monitoring, and providing feedback [72,73] and informative content (ie, quizzes and factoids) [74]. Furthermore, 67% (2/3) of the studies focused also on health behaviors other than PA (Multimedia Appendix 4 [67-87]). The interventions tested in the included studies were perceived as engaging by the youth. Youth liked to play games that aimed to increase their PA level and evaluated apps or text messaging systems as easy to use and supportive in improving PA or lifestyle behaviors. Multiple studies (6/21, 29%) found that poor functionality and technical issues are considered a barrier for engagement with mobile interventions [67,68,75-77] and suggested to include technical improvements [76].

In the following section, all the design features are summarized, starting with the design feature researched most often. A detailed overview of the results is presented in Table 1. All tested design features (ie, features offered to participants via an app and actually tested) are outlined as an association between the particular design feature and engagement (either as self-perceived association in qualitative studies or as tested association in quantitative studies). In case the participants provided further suggestions on design features that might be included in a future version (ie, feature not tested or only hypothetical), it was explicitly referred to as a suggestion. The results section concludes with a subanalysis on BCTs and low-SEP subgroups.

**Table 1:** Design features and association with user engagement.

Details			Association (ie, tested)	Suggestion (ie, hypothetical)
**Interface aesthetics**				
	**Language**			
		PA^a^ and nutrition messages (ranging from information to quizzes)	[74]^b,c^ [67]^b,d^ [74]^b,c^	[67]^d^
		Familiar content	[74]^c,e^	N/A^f^
		Short messages	[78]^b,g^ [74]^b,c^	N/A
		Cheerful and personal tone	[78]^b,g^ [74]^b,c^	N/A
	**Visual**			
		Clearly presented text	[68]^b^	N/A
		Large text blocks and complicated language	[68]^e,g^	N/A
		Clear and realistic visualization (eg, infographics, figures, tables, pictures, symbols, and videos)	[79]^b,g^ [68]^b,g^	[68]^g^ [67]^d^ [76]^c^ [77]^d^
		Quality not reflecting reality	[70]^e,g^	N/A
	**Sound**			
		Sounds associated with arm movements or game activities	[75,80]^b,c^	N/A
		Sounds associated with every click	[81]^e,g^	N/A
		Monotone and robotic voice	[75]^c,e^	N/A
	**General interface**			
		Lack of sophistication	[75]^c,e^ [79]^e,g^	N/A
**Reinforcement**				
	**Rewards**			
		Tangible rewards (prize, medal, new Pokémon, and healthy real-world actions)	[75,82]^b,c^ [78]^b,g^	[83]^c^ [78]^g^
		Intangible rewards (achievement, encouragement, evolving Pokémon, and comparisons)	[70,81]^b,g^ [82,84]^b,c^	N/A
	**Messages**			
		Messages sent at random times, maximum frequency <2 times per day	[74]^b,c^	N/A
		Frequency and timing of messages	[78]^e,g^	N/A
		Receiving reminders	[79]^g,h^	N/A
	**Reinforcement**			
		Leaderboard	[76]^h,c^ [87]^h,d^ [85]^e,c^	N/A
**Navigation**				
	**PA instructions**			
		Instructions on PA (eg, video)	[67]^b,d^	[71]^g^ [86]^c^
		Textual and unclear instructions on physical missions	[71]^e,g^ [83,85,86]^c,e^	N/A
	**In-app instructions**			
		Clear and intuitive in-app instructions	[84]^b,c^	[80]^c^
		Written manual and unclear instructions	[78]^e,g^ [76]^c,e^	N/A
	**Layout**			
		Straightforward and simple layout	[84]^b,c^	[67]^d^ [83]^c^
		Scrolling to find information	[68]^e,g^	N/A
		Lack of logical flow between different modules	[68]^e,g^	N/A
	**Navigation**			
		Finding and recognizing items on a map	[70]^e,g^	N/A
		Difficulty reading the map	[76]^c,e^	N/A
		Controls reacting slowly	[78]^e,g^ [85]^c,e^	N/A
**Social**				
	**Multiplayer**			
		Socializing and multiplayer capabilities (eg, friends)	[80]^b,c^ [70,78,81]^b,g^	[76,80,82]^c^ [71]^d^
	**Social messages**			
		Sharing results and postings	[78]^b,g^ [83]^b,c^	N/A
		Social networking	[84]^c,e^	N/A
		Replying messages or chat	[74]^b,c^	[82]^c^
	**Competition**			
		Competing against classmates	[71]^g,h^ [82]^c,h^ [87]^d,h^	N/A
		Competing with friends	[87]^d,h^ [78]^b,g^	N/A
	**Cooperation**			
		Cooperation and togetherness	[82]^b,c^	[82]^c^
**Challenges**				
	**Types of challenges**			
		Searching for items (eg, QR codes, bombs, Pokémon, and tags)	[70,71,81]^b,g^ [82]^b,c^	N/A
		PA missions	[84]^b,c^ [69,71,78]^b,g^	N/A
	**Variety**			
		Repetitive game with lack of progression or clear end goal	[78]^e,g^	N/A
		Larger variety of actions (eg, special events, season-themed challenges, and new missions or minigames)	N/A	[78]^g^ [76,82]^c^ [77]^d^
	**Difficulty level**			
		Difficulty level of the challenges that suit a player’s skill level	[81]^b,g^	[80]^c^
	**Time limit**			
		Time given (to diffuse bombs)	[70]^g,h^	N/A
**Monitoring**				
	**Manual input**			
		Manual input of multiple activities or behaviors	[68,69]^e,g^	N/A
		Remembering multiple behaviors	[79]^e,g^	N/A
		Run logs and PA diary	[84]^b,c^ [67]^b,d^	N/A
	**Device**			
		Monitoring via phone (eg, SMS text messaging and smartphone)	[73]^b,d^ [71]^b,g^	N/A
		Monitoring via external sensor (eg, pedometer, wearables, and heart rate monitor)	[73]^b,d^ [71]^b,g^	N/A
		Monitoring with real-time information	[83]^b,c^ [77]^d,h^	N/A
	**Self-monitoring**			
		Self-reflection	[72]^c,h^	N/A
		Ability to track multiple behaviors	[79]^g,h^	N/A
**Goals**				
	**Self-set goals**			
		Personal, self-selected goals	[67]^b,d^ [78,79]^b,g^ [76]^c,h^	N/A
	**Type of goals**			
		Choosing between different types of goals	[84]^b,c^	N/A
		Goals helped to perform PA	[83]^b,c^ [67]^b,d^	N/A
	**Goals**			
		Goal reminders	[72,76]^b,c^	N/A
**Customization**				
	**Types of customization**			
		Ability to customize user account, own music, choosing the type of PA, and personal goals	[78,79]^b,g^ [83,84]^b,c^	[83]^c^
	**Avatars**			
		Avatars (and additional sports equipment)	[69]^b,g^	[69]^g^
**Feedback**				
	**Types of feedback**			
		Feedback message on goals, weekly PA, real-time information, and individualized feedback	[67]^b,d^ [83]^b,c^ [79]^b,g^ [77]^d,h^	N/A
	**Representation**			
		Feedback presented in graphs or via SMS text messages	[67,73]^b,d^	N/A
**PA input**				
	**PA input**			
		Variety of arm movements and physical exercise	[80]^b,c^ [71]^b,g^	N/A
		Running, PA, and FMS^i^ components	[70,81]^g,h^	N/A
**Narrative**				
	**Characters**			
		Variety of virtual components (eg, zombies), weapons, and ways to increase character abilities	[80,84]^b,c^	[78]^g^
	**Setting**			
		Realistic	[80]^b,c^	N/A
		More world to explore	N/A	[78]^g^
	**Story**			
		Complex story line	N/A	[70]^g^ [80,86]^c^
**Levels**				
	**Increasing levels**			
		Gradually increasing levels	[84]^b,c^	[69]^g^
**Credibility**				
	Messages originating from nutrition professionals		[74]^b,c^	N/A
**Other features**				
	**Push notifications**			
		Additional push notifications	N/A	[80]^c^
	**GPS**			
		Reduce GPS latency	N/A	[76]^c^

^a^PA: physical activity.

^b^Positive association between features and engagement.

^c^Qualitative studies, assessing self-perceived association.

^d^Quantitative studies, assessing association.

^e^Negative association between features and engagement.

^f^N/A: not applicable.

^g^Mixed methods studies.

^h^Positive and negative associations between features and engagement.

^i^FMS: fundamental movement skills.

### Interface Esthetics

Clear and short messages with positive and personal tone and relevant content were associated with engagement in youth [67,74,78]. Engagement was hampered by lack of sophistication in the interface (eg, game graphics, sound effects, and camera use) [75,79]. Youth preferred a clear and understandable layout [67,68,76] and text with clear headlines and a large font size supported with infographics, figures, and tables [68]. The design of progress graphs was identified as an important contributor to engagement in terms of readability [79]. Too much text or large text blocks, as well as heavy and complicated language, were considered to hamper usability [68]. Participants suggested to include more pictures, symbols, and videos to make the app more attractive [68]; improved or realistic visuals [67,76,77]; or general interfaces [76]. Youth liked sounds that were associated with their movements or game activities [75,80]. However, robotic and monotonic voices [75] or sound associated with clicks could negatively impact engagement [81].

### Reinforcement

Youth enjoyed winning tangible rewards [70,75,78,82] and suggested offering rewards (ie, in apps that have not introduced rewards earlier) [78,83]. Intangible rewards (eg, feeling of achievement [81] and encouragement [84]) were especially interesting for the youth. Messages that were sent at random times were preferred over messages sent at preset times. The preferred frequency of the messages was less than twice per day [74]. In the study by Martin et al [78], participants expressed concerns about the frequency and timing of messages. The timing should be appropriate, for example, not receiving messages at a moment when one is not able to act on the messages [74,78]. Mixed results with regard to leaderboards were noted. Youth enjoyed leaderboards when beating persons placed above them but they did not enjoy being at the top of the leaderboard. In addition, leaderboards could lead to too much competition that could result in teasing [76]. In another study, adolescents particularly indicated that they missed seeing the other player’s scores [85].

### Navigation

Instructions related on the one hand to instructions of the in-app functions and on the other hand to instructions related to PA activities. In both cases, well-outlined instructions could support engagement [67,84], and suggestions were made to design clear, simple, and intuitive instructions [80] with the addition of visual components [71,86]. Unclear instructions on requested movements [83,85,86] or unclear [76] textual [71,78] instructions could hamper engagement. The wish for clear instructions had been underlined by a straightforward and simple layout [67,68,83,84]. Working with a map was considered difficult [70,76]. Laine and Suk [70] reported that the quality of the map tile was perceived as poor, which made it difficult to identify one’s current location. In addition, Robertson et al [76] reported that children experienced difficulties in understanding the map representation in the game interface. Interestingly, both studies integrated the Google Maps (Google Inc) services, which hampered PA and interrupted engagement. To find and recognize targets, the map resolution and targets should be clear. Furthermore, controls facilitating navigation through the app were negatively associated with engagement in case they were reacting slowly [78,85].

### Social

Multiplayer gameplay was considered important for engaging, socializing [80,81], and playing with friends [70] and setting challenges for friends [78]. In different studies, multiplayer gameplay was suggested to include in future versions of the app [76,77,80,82]. However, competing with friends revealed mixed results. Martin et al [78] found a positive association with engagement [78], and as outlined previously, leaderboards could affect high competition (see *Reinforcement* section); however, when competition was compared with cooperation, the latter was preferred [71,82,87]. This was also quantitatively assessed by Nuijten et al [87], who found that intergroup competition (ie, collaboration within class and competing against other classes) increased engagement in comparison with intragroup competition (ie, individual competition). Competing with others was suggested by youth for future app versions [80]. Cooperation and togetherness were considered important during gameplay [82,87], and an extension to collaborate with others was suggested for future app versions [82]. Social interaction in terms of messages to the research team [74], postings [83], and sharing results [78] was considered to contribute to engagement. A chat function [82] or a social platform [80] was suggested for a future app version. Direito et al [84] described that children seldom or never used the social networking features and disliked sharing their runs or status updates.

### Challenges

Youth suggested increasing the variety of actions or (mini) games available, indicating that they liked a wide variety of challenges to choose from when playing a game [77,78]. In addition, the new content was considered important, for example, season-themed challenges or special events [76,78,82]. Youth liked activities organized around a hunt, including activities of finding and scanning objects [70,71,81,82] or missions, for example, PA-related workout missions [69,71,78,84]. Youth liked games that made a connection between energy expenditure in real life and the energy level in a game [78]. Repetitive challenges with a lack of progression or clear end goals negatively impacted engagement [78]. The difficulty level of the challenges needed to fit the users’ level [80,81], as challenges that were not adjusted to a younger age appeared to negatively impact young kids’ enjoyment [81]. This finding was supported by the study done by Arteaga et al [80], in which children suggested including multiple challenges that fit the different levels of individual players. The study by Laine and Suk [70] reported mixed results regarding a time limit paired with challenges.

### Monitoring

Manual input hampered engagement [68,69,79], although youth liked to make use of logbooks [84] and diaries [67]. Using devices [71,73] to track real-time information was appreciated and perceived as useful [77,83]. Youth endorsed the self-monitoring function but had complaints relating to discomfort while wearing the monitoring device [72]. The accelerometer that was used in this study was not part of the intervention but was used to measure the PA outcome. The perceived discomfort might have influenced the use of the intervention. Including the function to track multiple health behaviors, on the one hand, engaged youth but, on the other hand, challenged them by the need to remember multiple behaviors [79].

### Goals

Self-set goals were considered important [67,76,78,79]. However, choosing a goal was considered cognitively challenging [76]. Specific goals on PA or different types of goals were considered to contribute to engagement [67,83,84]. The feeling of achieving a goal contributed to enjoyment [76]. Reminders to set goals were considered useful [72].

### Customization

Youth liked to customize their accounts (eg, removing features such as friends, goal-setting, and training plans) [79,83]; choose the type of PA they wanted to perform [83]; sort their personal goals [78]; and listen to their own music [84]. Youth in general suggested including the possibility of customizing one’s account [83].

Youth liked to engage with avatars [69], and children (10-12 years old) suggested including dragons (35%), followed by a dinosaur (25%), and suggested to upgrade their avatar with personal equipment [69].

### Feedback

Participants considered regular feedback on achievements and goals [67], which is individualized and in real time to contribute to engagement [79,83]. However, feedback based on dynamic tailoring did not reveal to contribute to overall engagement but contributed to narrative sensation (ie, feeling of presence) [77]. Feedback that was shared via SMS text messaging and presented in graphs was highly valued [67,73].

### PA Input

Youth enjoyed upper-body movements [80] compared with full-body movement such as running. However, requiring running to proceed in the game achieved mixed results (ie, facilitating and hampering) on engagement [70,71,81].

### Narrative

Youth liked the addition of a narrative, including a variety of characters [78,80,84] and settings [78] that are realistic [80], and the addition of a (complex) story, which was suggested by various studies [70,80,86].

### Levels

Gradually increasing levels [84] were considered important, and higher upgrade levels, referring to unlocking new levels as the player proceeds, were suggested to be added [69].

### Credibility

Youth found it important to receive information from a credible source as nutrition professionals [74].

### Other Features

Youth suggested including additional push notifications [80] and reducing the GPS latency [76].

### Included BCTs

In total, 19% (4/21) of the studies did not include any reference to BCTs [68,71,82,86]; 19% (4/21) of studies did only include a reference to BCTs in the results [70,73,74,80]; and 33% (7/21) of studies did include a reference to BCTs in the methods and results; however, they did not particularly refer to it as BCT [67,69,75,77,78,84,87]. Furthermore, 29% (6/21) of studies included a particular reference to BCTs and included the outline of the BCT in the methods and results [72,76,79,81,83,85]. In general, 57% (12/21) of studies included theory in the development of the app (4/21, 19% excluded as apps developed for commercial purposes). In addition, 81% (17/21) of studies included several BCTs (ranging from 2-10), which were partly related to the design features outlined in Table 1. The particular BCTs were not adopted in Table 1, as they have been translated into design features. The BCTs that were most often mentioned were feedback (11/21, 52%), social comparison (11/21, 52%), goal-setting (10/21, 48%), rewards (10/21, 48%), monitoring (9/21, 43%), and encouragement (6/21, 29%).

### Subanalysis SEP

In total, 29% (6/21) of the studies (all from countries that form the Global North) included a substantial target population of low SEP [72,75-77,86,87]; however, 33% (2/6) of studies eventually included a large percentage of high-SEP children (34% to 75%) [72,76]. In only 33% (2/6) of studies [75,76], the SEP was indicated as a direct study aim, and in 33% (2/6) of studies [77,86], a mix in SEP was desired. Low SEP was defined differently (ie, neighborhood site, family income, family affluence, parents’ education level, and youth education level), which made it difficult to compare studies, and engaging design features were not presented for different SEP categories. Therefore, subanalysis of the different design features could not be conducted.

## Discussion

### Principal Findings

This systematic review presents the results of 21 studies that assessed the associations between features and engagement with mHealth PA interventions. According to our knowledge, this is the first mHealth intervention review that focused on PA and youth, with an additional focus on low-SEP background.

The results showed that various design features, such as a clear interface; rewards; multiplayer game mode; facilitation of social interaction; variety of challenges with personalized difficulty level; self-monitoring options; and a variety of customization options among others, including self-set goals, personalized feedback, progress, and a narrative, were positively associated with engagement. In contrast, various features, such as sounds, leaderboards, competition, instructions, timing of messages or notifications, maps, or self-monitoring facilitated by manual input, that have been negatively associated with engagement need to be carefully considered while designing mHealth PA interventions. In addition, technical functionality can be considered as a prerequisite for engagement.

When comparing the results with those of research in adults, several features are shared, such as a simple and structured interface, tailored and positive feedback on PA levels [43,44,89,90], progress [90], well-designed reminders (eg, timing and frequency of notifications) [26,43,44,89], rewards [43,44,49,91-93], self-monitoring [26,43,44], goal-setting, [43,44], clear navigation [43,44], accurate tracking function [43], personalization (eg, goals) [94], and the offer of a variety of features [43,95]. Technical difficulties were also negatively influencing engagement in adults [43,89].

Several features that have been in particular noted for adults, yet not in this review on youth, are credential sources, adequate privacy settings [43], and content preventing the occurrence of surprises [26,43]. Sharing accomplishments via social media was considered less engaging in adults [90] than in youth. Although the studies among youth provided mixed results on competition, literature on adults suggests including competition [44,90], leaderboards [91], and hierarchical status (eg, progressive status report) [91]. In gamification research, leaderboards are considered the most common feature implemented (alongside points, goals, and progress) [96,97], and this seems to be considered with care when addressing youth for PA promotion. In studies including adults, no direct links with narratives, avatars, a hunt, difficulties with manual input, sounds, or virtual location maps were found. Research on using these design features for interventions in youth is limited, according to the results of this review.

This review focused on design features that have been tested by youth and included hypothetical or suggested features of the same studies but did not extend the search to studies that only provided ideas or suggestions that have not been tested (ie, cocreation study on app development without user-testing). Earlier studies have indicated that what users may request and what they actually engage with in practice do not have to match [44,98]. In the studies in this review, several features were tested and included as further suggestions, which may underline the importance of the match between what youth would like to have included and what they actually engage with. The following results are in line with existing research on app development (ie, no user-testing) among youth: (1) clear user interface esthetics (ie, youthful visuals) [99,100]; (2) rewards, referring to a fair reward system [100,101], rewards that progressively increase [102], and social rewards (ie, storybooks with interactive questions) [103]; and (3) multiplayer mode, facilitating competition [55,100] and comparison (eg, leaderboards) [55], as well as cooperation [103,104]. Several features that have been suggested and tested in the studies included in this review were not found in existing research, and these include (1) chat functions (although with mixed results), (2) increasing difficulty level, (3) customizing user content, and (4) adding a variety of avatars and characters. In addition, several features have been suggested but have not been tested, and these include (1) meaningful information [99,100], including PA-related tips and plans [101], and (2) variety and content updates [102].

Features that have been solely suggested in this review (ie, not tested) relate to (1) adding video and moving figures that navigate the PA and app instructions, (2) adding a storyline, and (3) including additional push notifications. Other research suggests that a clear navigation that is self-explanatory is important [99], yet that notifications evoke mixed results on engagement [101]. Features that have been suggested in other research, but not in this review, include (1) in-app events [104] and (2) GPS and map editors [105,106]. This review contributes to the body of research and highlights that although maps can be suggested by youth, they can be very challenging when applied in practice, risking hampering engagement. Additional features that especially could hamper engagement include a small number of available minigames [106], no instant feedback [102] or personalized feedback by email [101], lengthy texts, and difficult navigation [99]. Future studies are needed to conduct experimental testing on features that yield inconclusive evidence so far among others competition, leaderboards, and notifications, as it may be possible that the results may differ for different subgroups of youth. We recommend that designers and health researchers consider designing a clear interface and an appropriate and fair reward system, enabling social interaction, and providing a variety of content, which is preferably customizable.

Earlier research indicates how a variety of design features are designed and implemented in mobile apps [65,107]. This is also indicated in other research outlining the challenges of how BCTs are operationalized [65,108-110]. In other words, the content or the active ingredient of the intervention may still differ by how it is delivered, in which context, or in which combinations (ie, design features, including gamification elements and BCTs) it is applied [111]. An ontology, as proposed by West and Michie [112], and tools such as SciModeller that integrate multiple pieces of empirical data [113] can eventually contribute to this knowledge and help researchers and mHealth developers apply and build on this knowledge. This review focused on whether the app design has been informed by or has been based on relating BCTs or behavioral theories and concluded that BCTs are often not outlined in detail, and there is no particular translation of BCTs to app content. This review only included 33% (7/21) of studies that made a clear translation from a particular BCT to a design feature. A scoping review not only summarized the challenges in mHealth design in translating BCTs to an mHealth feature but also raised challenges with regard to integrating ideas from different perspectives (ie, BCTs, user needs, and stakeholder views), which can result in conflicting ideas [110]. Existing research on mHealth, focusing on app features, BCTs, or both, points to the challenge of mapping which features or BCTs work in isolation or in combination with others [47,114]. Furthermore, a wide applicability exists with regard to creating design features that are often designed from scratch. In light of the Open Digital Health initiative [115], we recommend making mHealth apps and data accessible to be able to reuse or continue working on existing design features that have been proven to be effective for engagement. By this, we can start mapping working design features for different user groups in different contexts [115,116].

Different systematic reviews focusing on the effectiveness of mHealth interventions have also focused on BCTs. However, a large number of the reviews map the number of BCTs that have been included in an intervention [42,117] or the BCTs that have been included [42,117]. However, there is dearth of research that identifies the individual or interaction effects of design features or BCTs in mHealth interventions among youth. Several studies have identified that modeling is an effective BCT for children. With regard to adolescents, providing consequences for behavior, providing information on others’ approval, facilitating intention formation, self-monitoring, using behavioral contract [118], and providing individualization support [25] were positive predictors of PA effect size. Providing instructions was negatively predictive of study effect size [118]. In future reviews, it would be interesting to map the similarities and differences between design features that contribute to engagement in the mHealth intervention (ie, microengagement) and BCTs that contribute to the behavioral change effectiveness (ie, macroengagement) to identify effective design features [40]. However, limited BCTs are researched in effectiveness studies, especially among youth [119]. Therefore, more individual studies are needed before this comparison can be made. Future studies may, therefore, test the effectiveness of individual BCTs and combinations of BCTs on behavioral changes in factorial designs [120] in youth. Future studies should also consider testing both microengagement and macroengagement in the same mHealth intervention [40] to better inform how to increase engagement in the mHealth intervention (ie, microengagement) and translate this to behavioral change, which can be engaged in the long term in the absence of any intervention (ie, macroengagement).

This review underlines that research addressing youth from low-SEP families is very limited with regard to engagement in mHealth apps. Although it is often argued that apps can be a suitable platform to reach diverse groups of youth, it is striking that only 7 studies have been identified that aim to involve youth from different SEP backgrounds. Unfortunately, it was not possible to conduct a subanalysis because the features were not directly linked to the SEP position. Studies that succeeded in addressing a large percentage of children with low SEP recruited participants via schools [49,77,86]. From this review, it cannot be directly derived which recruitment strategy leads to lower percentages of children with low SEP, as especially, the measures of low SEP differ greatly. We advocate for more research that includes youth from low-SEP families early in the development phase and user-testing and suggest recruiting youth from low-SEP background via schools. Further research in adults suggested that personal contact between study staff and participants is essential and that community sites (comparable with school settings) created a sense of community and support [121]. Thereby, youth who have the potential to book the greatest health gains (ie, often youth with low-SEP or low-PA levels) are addressed appropriately, which may contribute to reducing health inequalities and the digital divide [44]. A scoping review highlighted the need to further investigate user engagement studies in low-SEP groups and called for future in-depth formative studies [95]. Existing research indicates that multimedia, personalization, variation, and gamification [95], such as competition [116], can contribute to engagement in mHealth apps among young adults with low-SEP backgrounds. We advocate to start testing these features in youth with low SEP.

In this review, we identified a heterogeneity of engagement measures, which has also been identified in earlier reviews [55,60,114,122,123]. In addition, several measures only reflect one construct of engagement and do not measure the multidimensional concept of engagement [55]. Furthermore, research studies included in this review did not distinguish between different features that can be considered in different stages of engagement. Research suggests that engagement is a dynamic process rather than a state, although it is often measured as a state (ie, 1 postintervention questionnaire on engagement instead of cyclic measurement) [124]. On the basis of earlier research, it may however be stated that features such as a clear interface are especially important for the initial stage of engagement (ie, attention grabbing) [124]. Features that sustain engagement may relate to social interaction, a variety of challenges with personalized difficulty level, self-monitoring and customization options, and narratives [77]. Disengaging may relate to certain types of sounds, leaderboards, instructions, messages or notifications, competition, and self-monitoring facilitated by manual input. Features that reengaged or nonengaged users have not been identified in this review. However, for example, Janko et al [69] discussed unlockable avatars and upgrading levels as possible features that may contribute to reengagement. Research on young adults indicated that users did not engage (ie, nonengagement) owing to low uptake of the intervention among peers [49]. The reasons for disengagement mentioned in this review relate to factors outside the mHealth intervention (eg, holidays, competing after-school activities, weather, school policies, and unstructured or leisure settings) [76,77,81,83,87], yet nonadherence has not been linked to design features. Future research is needed to identify particular features in different stages of engagement to grasp engagement as a multidimensional and dynamic process [124]. Thereby, designers can improve design features according to the stage of engagement (eg, include particular features to reengage users and prevent them from sustained disengagement). Furthermore, engagement may also be related to the setting in which the mHealth tool is implemented. The activity that competes with the mHealth tool is central. Earlier research indicated that pupils tend to choose to engage with an mHealth tool in order not to participate in class in a school setting [125]. In comparison, mHealth tools are often challenged in leisure time, and the competition with other apps or leisure activities is central [126,127]. Future studies should focus on the implementation of mHealth tool and investigate differences in engagement in either voluntary versus more obligatory settings.

In terms of comparability, the Persuasive Systems Design (PSD) model could have helped map the different design features. PSD is often used in research to outline persuasive design elements. The model maps different persuasive design elements in primary task support, dialogue support, system credibility support, and social support. When comparing the PSD model with the coding scheme applied in this systematic review, a number of similarities can be identified. In terms of primary task support, navigation (PSD model: tunneling), personalization, and self-monitoring are identified. In terms of dialogue support, rewards, messages (PSD model: reminders), and interface aesthetics (PSD model: liking) can be identified. Liking is very broadly defined as “visually attractive” in the PSD model and has been criticized in earlier research [128]. It finds more detail in the coding of our systematic review (ie, interplay of interface elements such as sounds, visuals, and language). In terms of system credibility support, only credibility (PSD model: expertise) can be identified. In terms of social support, reinforcement (PSD model: social comparison), social messages (PSD model: social facilitation), cooperation, and competition can be identified. Design features that are difficult to code in terms of the PSD model are challenges, levels, feedback, avatars, and narratives and are predominately game elements and refer to achievement- and immersion-based features [96]. These have also been identified in earlier research, distinguishing their research from the PSD model [129,130]. On the one hand, this emphasizes the challenge to identify and apply a complete list of design elements. As a proposed solution, Geuens et al [128] created a website that derived from the PSD model and mapped a working list of adaptable features. However, it should be noted that this list is currently not complete in terms of gamification elements. On the other hand, the large number of similarities between the PSD model and the coding frame of this systematic review suggest that various research studies may have found (to a certain degree) a consensus on design features. A revision of the PSD model, adapted by, for example, gamification elements, could help create an updated list of design features that are widely applicable to designers and researchers.

### Strengths and Limitations

As identified in an earlier review [55], this review included a larger number of studies reporting the foremost positive associations, which may bias the overview in features that are either unrelated or negatively associated and may not be reported (ie, publication bias). In addition, the strength of associations has not been included in the individual studies, owing to a lack of high-quality experimental studies. In addition, heterogeneity among studies with regard to engagement measures was identified, making direct comparison challenging; this has also been identified in earlier studies [114]. This systematic review maps a large number of features that are associated with engagement individually. The interaction of different features and their effect on engagement is still not researched and needs to be considered in future engagement studies, for example, experimental factorial designs [41]. Individual studies included small sample sizes, and only a small number of studies focused on low SEP. Using different SEP measures and mixing them with high-SEP measures made it challenging to compare studies and prevented us from conducting a subanalysis based on the SEP background. Therefore, the findings might not be transferable to low-SEP groups and this underlines the need for future research. In addition, the variability in SEP measures made it difficult to standardize SEP thresholds and compare between countries, as youth from a low-SEP background may not be equivalent in terms of studies and countries, and disparity occurs even in different parts of one country. Studies that included SEP measures were exclusively from countries of the Global North, emphasizing that in general, a minority of studies focused on the Global South (4/21, 19%), and none focused on SEP. As the search was limited to English, this review may not have included all available and relevant research on this topic. Earlier research outlines the differences in the effectiveness of mHealth intervention on behavioral outcomes and engagement among very young children and older children. Research further suggests that preferences for design features may differ between different age groups of youth. This systematic review did not cluster for different age groups because of the limited number of studies that, for example, focus on the very young children (aged <4 years) [25]. More individual studies are needed among very young children to identify whether age differences exist. Earlier research suggests that gender differences in games may exist [131-137]. None of the included studies in this systematic review distinguished between gender for design features in mHealth interventions. In future research, it can therefore be considered to investigate possible differences in future user-testing. The quality of the studies was mixed, indicating in general that study designs with regard to measuring engagement need to evolve. Especially, studies that focus on engagement as a secondary outcome need to operationalize engagement clearly and report the methods transparently. In this review, no validated design feature taxonomy has been used for coding, although it was based on existing overviews of design features [114]. In this review, all study designs were included, and this provided a comprehensive overview of relating research on engagement and triangulated evidence. This is the first review identifying mHealth design features among youth with the aim of promoting PA. This review was based on the PRISMA guidelines and succeeded in providing a coherent overview of all nuances relating to a good quality assessment and high interrater reliability.

### Conclusions

The results indicate that a clear interface; an appropriate and fair reward system; social interaction; and a variety of app content (ie, missions, content, and characters) that are preferably customizable can contribute to engagement in mHealth PA interventions for youth. Design features, such as sounds, competition, instructions, notifications, virtual maps, or self-monitoring facilitated by manual input, that were negatively associated with engagement need to be carefully considered when designing mHealth PA interventions. In addition, technical functionality can be considered a prerequisite for engagement. Research addressing youth from low-SEP families is very limited with regard to engagement in mHealth apps, and design features often lack a sufficient degree of operationalization based on behavioral change theories or techniques. Future studies are needed to further test design features in youth, particularly youth from low-SEP families, and to evolve engagement measures.

## Appendix

Multimedia Appendix 1 PRISMA (Preferred Reporting Items for Systematic Reviews and Meta-Analyses) checklist.

Multimedia Appendix 2 Full search strategy.

Multimedia Appendix 3 Quality check Mixed Method Assessment Tool.

Multimedia Appendix 4 Data extraction of the studies included in the systematic review.

## Abbreviations

BCT: behavior change technique
mHealth: mobile health
MVPA: moderate- to vigorous-intensity physical activity
PRISMA: Preferred Reporting Items for Systematic Reviews and Meta-Analyses
PROSPERO: International Prospective Register of Systematic Reviews
PSD: Persuasive Systems Design
SEP: socioeconomic position
