# Supplementary material for: Design Features Associated With Engagement in Mobile Health Physical Activity Interventions Among Youth: Systematic Review of Qualitative and Quantitative Studies
Source: JMIR Mhealth Uhealth. 2023 Mar 6;11:e40898. doi: 10.2196/40898 (PMC10028523; doi:10.2196/40898)
Supplement: Multimedia Appendix 2 [file mhealth_v11i1e40898_app2.docx]

**Multimedia Appendix 2: Literature Search Strategies**

**Datum search: 02-06-2021**

**Updated search: 24-06-2022**

**Scopus**

| **Concept** | **Search term** | **Hits** | **24-06-2022**  **(limited to years 2021-present)** |
| --- | --- | --- | --- |
| #1 Engagement | TITLE-ABS-KEY ( engagement  OR  enjoyment  OR  immersion  OR  flow  OR  transportation  OR  involvement  OR  presence  OR  attention  OR  interest  OR  affect  OR  motivation  OR  pleasure  OR  adherence  OR  attrition  OR  preference  OR  acceptability  OR  experience  OR  satisfaction  OR  playability  OR  usability  OR  usage ) | 15,476,849 | 1,546,066 |
| #2 mHealth | TITLE-ABS-KEY ( mhealth  OR  m-health  OR  "mobile health"  OR  ehealth  OR  e-health  OR  "electronic health"  OR  app  OR  application*  OR  game  OR  exergame  OR  gamif*  OR  smartphone  OR  "smart phone"  OR  "mobile device"  OR  mobile-phone  OR  mobilephone  OR  iphone  OR  i-phone  OR  ipad  OR  i-pad  OR  android  OR  "cell phone"  OR  cellphone  OR  tablet  OR  smartwatch  OR  "smart watch"  OR  sensor  OR  wearable  OR  "activity tracker" ) | 8,203,222 | 886,830 |
| #3 Physical Activity | TITLE-ABS-KEY ( "physical activity"  OR  exercise*  OR  sport  OR  "physical fitness"  OR  "active transport"  OR  "leisure activity" ) | 1,085,019 | 113,588 |
| #4 Youth | TITLE-ABS-KEY ( youth  OR  child  OR  adolescent  OR  adolescence  OR  kid  OR  teen  OR  teenager  OR  puber  OR  puberty  OR  youngster  OR  junior ) | 4,588,135 | 308,434 |
| #1 AND #2 AND #3 AND #4 | ( TITLE-ABS-KEY ( engagement  OR  enjoyment  OR  immersion  OR  flow  OR  transportation  OR  involvement  OR  presence  OR  attention  OR  interest  OR  affect  OR  motivation  OR  pleasure  OR  adherence  OR  attrition  OR  preference  OR  acceptability  OR  experience  OR  satisfaction  OR  playability  OR  usability  OR  usage ) )  AND  ( TITLE-ABS-KEY ( mhealth  OR  m-health  OR  "mobile health"  OR  ehealth  OR  e-health  OR  "electronic health"  OR  app  OR  application*  OR  game  OR  exergame  OR  gamif*  OR  smartphone  OR  "smart phone"  OR  "mobile device"  OR  mobile-phone  OR  mobilephone  OR  iphone  OR  i-phone  OR  ipad  OR  i-pad  OR  android  OR  "cell phone"  OR  cellphone  OR  tablet  OR  smartwatch  OR  "smart watch"  OR  sensor  OR  wearable  OR  "activity tracker" ) )  AND  ( TITLE-ABS-KEY ( "physical activity"  OR  exercise*  OR  sport  OR  "physical fitness"  OR  "active transport"  OR  "leisure activity" ) )  AND  ( TITLE-ABS-KEY ( youth  OR  child  OR  adolescent  OR  adolescence  OR  kid  OR  teen  OR  teenager  OR  puber  OR  puberty  OR  youngster  OR  junior ) ) | 5,007 | 1,015 |

**Datum search: 02-06-2021**

**Updated search: 24-06-2022**

**EBSCOhost - Medline**

| **Concept** | **Search term** | **Hits** | **24-06-2022** |
| --- | --- | --- | --- |
| S1 Engagement | engagement or enjoyment or immersion or flow or transportation or involvement or presence or attention or interest or affect or motivation or pleasure or adherence or attrition or preference or acceptability or experience or satisfaction or playability or usability or usage | 5,975,700 | 447,040 |
| S2 mHealth | mHealth or m-health or “mobile health” or eHealth or e-health or “electronic health” or app or application* or game or exergame or gamif* or smartphone or “smart phone” or “mobile device” or mobile-phone or mobilephone or iPhone or i-phone or iPad or i-pad or android or “cell phone” or cellphone or tablet or smartwatch or “smart watch” or sensor or wearable or “activity tracker” | 1,661,879 | 189,530 |
| S3 Physical Activity | “physical activity” or exercise* or sport or “physical fitness” or “active transport” or “leisure activity” | 649,197 | 60,515 |
| S4 Youth | Youth or child or adolescent or adolescence or kid or teen or teenager or puber or puberty or youngster or junior | 3,527,318 | 148,758 |
| S1 AND S2 AND S3 AND S4 | S1 AND S2 AND S3 AND S4 | 2,838 | 433 |

**EBSCOhost – APA PsychINFO**

| **Concept** | **Search term** | **Hits** | **24-06-2022** |
| --- | --- | --- | --- |
| S1 Engagement | engagement or enjoyment or immersion or flow or transportation or involvement or presence or attention or interest or affect or motivation or pleasure or adherence or attrition or preference or acceptability or experience or satisfaction or playability or usability or usage | 1,904,392 | 65,482 |
| S2 mHealth | mHealth or m-health or “mobile health” or eHealth or e-health or “electronic health” or app or application* or game or exergame or gamif* or smartphone or “smart phone” or “mobile device” or mobile-phone or mobilephone or iPhone or i-phone or iPad or i-pad or android or “cell phone” or cellphone or tablet or smartwatch or “smart watch” or sensor or wearable or “activity tracker” | 290,357 | 10,594 |
| S3 Physical Activity | “physical activity” or exercise* or sport or “physical fitness” or “active transport” or “leisure activity” | 165,181 | 5,747 |
| S4 Youth | Youth or child or adolescent or adolescence or kid or teen or teenager or puber or puberty or youngster or junior | 1,174,616 | 29,880 |
| S1 AND S2 AND S3 AND S4 | S1 AND S2 AND S3 AND S4 | 1,867 | 76 |

**EBSCOhost – Psychology and Behavioural Sciences collection**

| **Concept** | **Search term** | **Hits** | **24-06-2022** |
| --- | --- | --- | --- |
| S1 Engagement | engagement or enjoyment or immersion or flow or transportation or involvement or presence or attention or interest or affect or motivation or pleasure or adherence or attrition or preference or acceptability or experience or satisfaction or playability or usability or usage | 292,238 | 16,553 |
| S2 mHealth | mHealth or m-health or “mobile health” or eHealth or e-health or “electronic health” or app or application* or game or exergame or gamif* or smartphone or “smart phone” or “mobile device” or mobile-phone or mobilephone or iPhone or i-phone or iPad or i-pad or android or “cell phone” or cellphone or tablet or smartwatch or “smart watch” or sensor or wearable or “activity tracker” | 51,237 | 2,575 |
| S3 Physical Activity | “physical activity” or exercise* or sport or “physical fitness” or “active transport” or “leisure activity” | 31,552 | 2,130 |
| S4 Youth | Youth or child or adolescent or adolescence or kid or teen or teenager or puber or puberty or youngster or junior | 242,427 | 10,339 |
| S1 AND S2 AND S3 AND S4 | S1 AND S2 AND S3 AND S4 | 263 | 23 |
